# The Chemical Profiling of Essential Oils from Different Tissues of *Cinnamomum camphora* L. and Their Antimicrobial Activities

**DOI:** 10.3390/molecules26175132

**Published:** 2021-08-24

**Authors:** Darbin Kumar Poudel, Anil Rokaya, Pawan Kumar Ojha, Sujan Timsina, Rakesh Satyal, Noura S. Dosoky, Prabodh Satyal, William N. Setzer

**Affiliations:** 1 Analytica Research Center, Kirtipur, Kathmandu, Nepal; darkwine51@gmail.com (D.K.P.); arokaya@aromaticplant.org (A.R.); pojha@aromaticplant.org (P.K.O.); stimsina@aromaticplant.org (S.T.); rsatyal@aromaticplant.org (R.S.); 2 Aromatic Plant Research Center, 230 N 1200 E, Suite 100, Lehi, UT 84043, USA; ndosoky@aromaticplant.org (N.S.D.); wsetzer@chemistry.uah.edu (W.N.S.); 3 Department of Chemistry, University of Alabama in Huntsville, Huntsville, AL 35899, USA

**Keywords:** camphor, safrole, enantiomeric distribution, antibacterial, antifungal

## Abstract

*Cinnamomum camphora* L. is grown as an ornamental plant, used as raw material for furniture, as a source of camphor, and its essential oil can be used as an important source for perfume as well as alternative medicine. A comparative investigation of essential oil compositions and antimicrobial activities of different tissues of *C. camphora* was carried out. The essential oils were extracted by hydrodistillation with a Clevenger apparatus and their compositions were evaluated through gas chromatography-mass spectrometry (GC-MS), enantiomeric composition by chiral GC-MS, and antimicrobial properties were assayed by measuring minimum inhibitory concentrations (MICs). Different plant tissues had different extraction yields, with the leaf having the highest yield. GC-MS analysis revealed the presence of 18, 75, 87, 67, 67, and 74 compounds in leaf, branch, wood, root, leaf/branch, and leaf/branch/wood, respectively. The significance of combining tissues is to enable extraction of commercial quality essential oils without the need to separate them. The oxygenated monoterpene camphor was the major component in all tissues of *C. camphora* except for safrole in the root. With chiral GC-MS, the enantiomeric distributions of 12, 12, 13, 14, and 14 chiral compounds in branch, wood, root, leaf/branch, and leaf/branch/wood, respectively, were determined. The variation in composition and enantiomeric distribution in the different tissues of *C. camphora* may be attributed to the different defense requirements of these tissues. The wood essential oil showed effective antibacterial activity against *Serratia marcescens* with an MIC of 39.1 μg/mL. Similarly, the mixture of leaf/branch/wood essential oils displayed good antifungal activity against *Aspergillus niger* and *Aspergillus fumigatus* while the leaf essential oil was notably active against *Trichophyton rubrum. C. camphora* essential oils showed variable antimicrobial activities against dermal and pulmonary-borne microbes.

## 1. Introduction

Essential oils are natural, complex, volatile chemical admixtures of an aromatic odor, extracted as secondary metabolites. Different types of essential oils have their applications in the pharmaceutical and flavoring industries. Consumption of essential oils has risen day by day either directly or indirectly because of consciousness in personal health and hygiene, and as alternative medicines [1]. Bacteria and fungi are encountered everywhere in the biosphere due to their metabolic ability and are easily grown under a wide range of environmental conditions; many are known to be pathogenic [2]. Essential oils have often been used in lieu of synthetic chemicals to counter microbial attacks that cause food spoilage, or infections. The available synthetic flavoring and antimicrobial chemicals can, however, be toxic and detrimental to our health if they exceed the prescribed limit of consumption [3]. Therefore, essential oils could be alternative solutions to ensuring food safety, retention of their nutritional value and quality, as well as eliminating human health risk. Some components from different plant essential oils are legally registered flavoring agents in foodstuffs because oxygenated monoterpenes display promising antimicrobial activity [4,5,6,7].

Different parts (roots, wood, branch, and leaf) of *Cinnamomum camphora* L. are abundant in essential oils, which have linalool, 1,8-cineole, and camphor as major components. Essential oils from *C. camphora* have chemotypes such as *iso*-borneol, camphor, 1,8-cineole, linalool, and borneol types [8,9,10]. Biosynthesis of secondary metabolites and their proportions in essential oils lead to variations in composition due to environmental factors (seasonal variation, geographic variation, light availability, herbivory and microbial infection, pH of the soil) pollution, herbicide and pesticide use, and anthropogenic behavior [11]. Due to its diverse climate, geology, and topographical area, Nepal’s biodiversity is a repository of secondary metabolites in essential oils. In Nepal, the chemotype of *C. camphora* essential oil is camphor, and its diverse bioapplication has been explored [12]. It has very significant prospective nutraceutical and pharmaceutical applications [13]. Oxygenated monoterpene chemotypes of *C. camphora* essential oil have multifunctional bioapplications such as antibacterial activities [14], antifungal activities [15], insecticidal activities [16], anti-inflammatory activities [17], and so on. These activities are related to the lipophilic nature of secondary metabolites in essential oils [18] and may act either by synergistic or antagonistic effects [12,19]. 

In this work, the chemical and enantiomeric compositions of the essential oils from different tissues (root, wood, branch, and leaf) of *C. camphora* were analyzed and compared. In addition, their effectiveness against dermal and pulmonary-borne microbes of the essential oils from different tissues of *C. camphora* L. was explored.

## 2. Results and Discussion

### 2.1. Isolation and Yields of Essential Oils and Yields

The highest essential oil yield was observed for the leaf (2.67%), whereas the lowest yield was from the wood (1.0%) of *C. camphora*. A slightly higher extraction yield was observed compared to previous reports of *C. camphora* leaf essential oil [12]. The yield of extraction depends on different factors such as geographical variation, harvesting time, extraction methods, extraction temperature and extraction time. The essential oil yields from different *C. camphora* tissues are shown in Table 1. 

### 2.2. Chemical Composition of Essential Oils

The individual chromatograms of *C. camphora* tissues are shown in Supplementary Figure S1–S6. In total, the presence of 18, 75, 87, 67, 67 and 74 compounds were identified in leaf, branch, wood, root, leaf/branch, and leaf/branch/wood, respectively. Relative percentages of the individual components of *C. camphora* essential oils are listed in Table 2. The predominant compound in the leaf oil was camphor (93.1%), followed by camphene (1.8%) and α-pinene (1.6%). Camphor (53.6%) was also the major component in the branch, followed by limonene (7.4%), α-pinene (6.9%), 1,8-cineole (3.7%), camphene (3.4%) and β-pinene (3.1%). The wood essential oil was rich in camphor (53.2%), 1,8-cineole (19.8%), α-terpineol (6.2%), and safrole (3.2%). In contrast, the root essential oil was composed of safrole (57.6%), 1,8-cineole (18.1%), camphor (11.8%), and α-terpineol (4.1%). The essential oil from a mixture of leaf/branch was rich in camphor (53.3%), α-pinene (8.2%), limonene (7.1%), camphene (3.9%), myrcene (3.7%), 1,8-cineole (3.2%), and β-pinene (3.2%). The major components in the essential oil from a mixture of leaf/branch/wood were camphor (59.5%), 1,8-cineole (6.2%), α-pinene (5.7%), and limonene (5.3%). The compound classes of the essential oils from leaf, branch, wood, roots, leaf/branch and leaf/branch/wood are presented in Table 2. Oxygenated monoterpenes accounted for the major proportion, which was dominated by camphor in all essential oils except for the phenylpropanoid, safrole, in the root.

### 2.3. Enantiomeric Composition of Essential Oil Terpenoids

In total, 12, 12, 13, 14 and 14 chiral terpenoid components were evaluated for their enantiomeric distributions in the branch, wood, root, leaf/branch, and leaf/branch/wood essential oils, respectively. Relative percentages of the levorotatory and dextrorotatory enantiomers for each of the chiral components in the essential oils are listed in Table 3. The biosynthesis of levorotatory and dextrorotatory compounds from any geographical location is almost constant and may be used for authentication of *C. camphora* essential oils. Camphor is the major oxygenated monoterpene and was (+)-camphor-predominant in our study. Similar enantiomeric distributions have been reported for *C. camphora* essential oil [15]. α-Thujene, sabinene, α-phellandrene, *cis*-sabinene hydrate, *trans*-sabinene hydrate, and β-caryophyllene were detected for the first time in this current study. β-Caryophyllene and sabinene were consistently present as dextrorotatory enantiomers. Interestingly, the wood and root essential oils showed contrasting type enantiomeric distributions for α-pinene and camphene. Likewise, the branch and wood essential oils showed oppositive enantiomeric relationships for α-thujene, borneol, and α-terpineol. Additionally, the branch and root essential oils showed contrasting distributions for α-thujene, α-pinene, camphene, borneol, and α-terpineol.

### 2.4. Antibacterial and Antifungal Activity

The essential oils of *C. camphora* have demonstrated a broad range of antimicrobial activities against different pathogens [12,20,21]. The minimum inhibitory concentrations (MICs) of the different *C. camphora* essential oil against a panel of bacteria and fungi are presented in Table 4 and Table 5, respectively. The wood essential oil showed good antibacterial activity against *Serratia marcescens*. In the wood oil, camphor, 1,8-cineole, α-terpineol, and safrole were the major components, and the observed activity of the wood oil against *S. marcescens* may be due to synergism among these and other constituents. Camphor, 1,8-cineole, α-terpineol, and safrole showed only marginal activity against *S. marcescens* (MIC = 312.5 μg/mL). A study suggested that a synergistic antimicrobial effect occurs between 1,8-cineole and camphor [22]. The essential oils of *C. camphora* demonstrated weaker antibacterial activities than those of the positive control, gentamicin (MIC < 19.5 μg/mL).

The essential oil from the mixture of leaf/branch/wood of *C. camphora* displayed good antifungal activity against *Aspergillus niger* and *Aspergillus fumigatus* (MIC = 78.1 μg/mL), while the leaf essential oil was notably active against *Trichophyton rubrum* with an MIC of 78.1 μg/mL. The essential oils of *C. camphora* demonstrated weaker antifungal activities than those of the positive control, amphotericin B (MIC < 19.5 μg/mL). Safrole alone was effective against *Aspergillus fumigatus*. However, the safrole-rich root essential oil showed no such effectiveness, which may be due to antagonistic effects among constituents. The antifungal mechanisms of activity of essential oils are not clearly understood. However, it has been postulated that the hydrophobic constituents either disrupt cytoplasmic membranes leading to cytoplasmic leakage, cell lysis, and ultimate death, or inhibition of sporulation [23].

## 3. Materials and Methods

### 3.1. Plant Material and Isolation of Essential Oils

The different fresh plant tissues of *C.* *camphora*, collected in May 2019 from Tribhuwan University, Kirtipur, Kathmandu, were cut into smaller pieces. For each plant tissue sample, a ratio of 1:3 plant sample and water was subjected to hydrodistillation using a Clevenger apparatus for 3 h. The essential oils were dried over anhydrous sodium sulfate and stored in bottles at 5 °C until further use. Essential oil yields from different tissues are summarized in Table 1.

### 3.2. Chemical Composition Analysis by Gas Chromatography/Mass Spectrometry (GC-MS)

The essential oils from *C. camphora* were analyzed by a Shimadzu GCMS-QP2010 Ultra with electron impact (EI) mode with 70 eV along with ZB-5MS capillary GC column. 40–400 *m/z* scan ranges with a scan rate of 3.0 scan/s. The column temperature was programmed at 50 °C for 2 min and then increased at 2 °C/min to a temperature of 260 °C. The carrier gas was helium with a column head pressure of 552 kPa and a constant flow rate of 1.37 mL/min. The injector temperature was kept at 260 °C. For each essential oil sample, 1:10 (*v/v*) solution in dichloromethane (DCM) was prepared and 0.3 μL was injected using a split ratio of 1:30. Identification of the individual components of the essential oils was determined by comparison of the retention indices and comparison of the mass spectral fragmentation patterns (over 80% similarity match) with those found in the MS databases using LabSolutions GCMS solution software version 4.45 (Shimadzu Scientific Instruments, Columbia, MD, USA) [24,25,26]. The relative percentages of the individual components are listed in Table 2.

### 3.3. Enantiomeric Analysis by Chiral Gas Chromatography-Mass Spectrometry (CGC-MS)

Enantiomeric analysis of *C. camphora* essential oil was carried out using a Shimadzu GCMS-QP2010S with EI mode (70 eV) having a Restek B-Dex 325 chiral capillary GC column, 40–400 *m/z* scan ranges with a scan rate of 3.0 scan/s. The column temperature was programmed at 50 °C, at first increased by 1.5 °C/min to 120 °C and then 2 °C/min to 200 °C. The final temperature of the column was maintained at 200 °C. The carrier gas was helium with a constant flow rate of 1.8 mL/min. For each essential oil sample, a 3% *w*/*v* solution in DCM was prepared, and 0.1 μL was injected using a split ratio of 1:45 [24,25,26]. The enantiomer percentages were determined from peak area. Identification of enantiomers was determined by comparison of retention times with authentic samples obtained from Sigma-Aldrich (Milwaukee, WI, USA). Enantiomeric distribution of chiral terpenoid components in *C. camphora* essential oils are listed in Table 3.

### 3.4. Antibacterial Screening

All tested bacteria were cultured on tryptic soy agar medium. A 5000-μg/mL solution of each essential oil was prepared in dimethyl sulfoxide (DMSO), and 50 μL diluted in 50 μL of cation-adjusted Mueller Hinton broth (CAMHB) (Sigma-Aldrich, St. Louis, MO, USA) was added to the top well of a 96-well microdilution plate. The prepared stock solution of essential oils was then serially two-fold diluted in fresh CAMHB to obtain final concentrations of 2500, 1250, 625, 312.5, 156.3, 78.1, 39.1 and 19.5 μg/mL. Freshly harvested bacteria with approximately 1.5 × 10^8^ CFU/mL final concentration (determined using the McFarland standard) were added to each well of 96-well microdilution plates that were incubated at 37 °C for 24 h. Gentamicin (Sigma-Aldrich, St. Louis, MO, USA) and DMSO were used as positive and negative controls, respectively [25,27].

Seven microorganisms were used to evaluate the antibacterial activities of the different tissues of *C. camphora* essential oils (five Gram-positive bacteria, *Bacillus cereus* (ATCC-14579), *Staphylococcus epidermidis* (ATCC-12228), *Propionibacterium acnes* (ATCC-11827), *Staphylococcus aureus* (ATCC-29213), and *Streptococcus pyogenes* (ATCC-19615), and two Gram-negative bacteria, *Serratia marcescens* (ATCC-14756) and *Pseudomonas aeruginosa* (ATCC-27853)), using the microbroth dilution technique. Antibacterial activities assessed by minimum inhibitory concentrations (MICs) are listed in Table 4. The microorganisms were purchased from ATCC (Lines 199–203), and cells harvested from freshly cultured plates were used for the assay.

### 3.5. Antifungal Screening

All tested fungi were cultured on yeast-nitrogen base growth medium (Sigma-Aldrich, St. Louis, MO, USA). Stock solutions (5000 μg/mL) of the essential oils were prepared in DMSO and diluted as above. The freshly harvested fungi, with approximately 7.5 × 10^7^ CFU/mL final concentrations, were added to each well of 96-well microdilution plates and were incubated at 35 °C for 24 h. DMSO and amphotericin B (Sigma-Aldrich, St. Louis, MO, USA) were negative and positive antifungal controls, respectively [25,28]. Seven fungal strains were used: *Aspergillus niger* (ATCC-16888), *Candida albicans* (ATCC-18804), *Microsporum canis* (ATCC-11621), *Trichophyton mentagrophytes* (ATCC-18748), *Aspergillus fumigatus* (ATCC-96918), *Microsporum gypseum* (ATCC-24102), and *Trichophyton rubrum* (ATCC-28188). All fungi were cultured on yeast malt agar and were harvested from a fresh culture in fresh yeast-nitrogen base growth medium (broth) added to each well. The antifungal activities (MICs) are listed in Table 5.

## 4. Conclusions

Different plant tissues of *Cinnamomum camphora* L. were collected from the Tribhuwan University area. The essential oils from these different tissues showed differences in chemical compositions, enantiomeric distributions, and antimicrobial activities. The yield of extraction varied depending upon the tissue used. The significance of combining tissues was to enable extraction of commercial quality essential oil without the need to separate them. However, combining tissues had low extraction yields compared to individual tissues. The oxygenated monoterpenoid camphor was the dominant component in all parts of *C. camphora* except for the root essential oil, which was rich in safrole. The data analysis in this study can used to create a benchmark for future *C. camphora* essential oil assessments, as well as authentication for adulteration or consumer safety. The wood essential oil showed the best antibacterial activity against *Serratia marcescens* among the tested bacterial strains with an MIC of 39.1 μg/mL. The leaf/branch/wood essential oil showed good antifungal activity against *Aspergillus niger* and *Aspergillus fumigatus,* while the leaf essential oil showed good antifungal activity against *Trichophyton rubrum* with an MIC of 78.1 μg/mL. *Cinnamomum camphora* L. and its essential oils can be used as important source antibacterial and antifungal agents.

## Figures and Tables

**Table 1:** Essential oil yields from different tissues of *Cinnamomum camphora* L.

*Cinnamomum camphora* L. Tissues	Yields
Leaf	2.67%
Branch	2.0%
Wood	1.0%
Root	1.8%
Leaf/branch (1:1 ratio)	2.17%
Leaf/branch/wood (1:1:1 ratio)	1.40%

**Table 2:** Essential oil compositions of *Cinnamomum camphora* L. (Tr indicates trace, <0.05%).

RRI	Compounds	*Cinnamomum camphora* L. Tissues Along with Relative Abundance (%)					
		Leaf	Branch	Wood	Root	Leaf/Branch	Leaf/Branch/Wood
921	Tricyclene	Tr	0.1	Tr	Tr	0.1	Tr
924	α-Thujene	0.1	0.5	0.2	0.1	0.5	0.4
931	α-Pinene	1.6	6.9	1.6	0.6	8.2	5.7
945	α-Fenchene	-	0.1	0.1	Tr	0.1	0.1
946	Camphene	1.8	3.4	0.9	0.2	3.9	2.9
967	Sabinene	0.1	0.9	1.4	1.0	1.0	1.0
974	β-Pinene	0.8	3.1	0.9	0.5	3.2	2.2
988	Myrcene	0.3	2.8	1.2	0.3	3.7	2.6
998	Octanal	-	Tr	Tr	Tr	Tr	Tr
999	δ-2-Carene	-	Tr	Tr	Tr	Tr	Tr
1002	α-Phellandrene	0.1	1.8	0.4	0.1	1.1	1.0
1008	δ-3-Carene	-	0.1	Tr	-	0.1	0.1
1014	α-Terpinene	Tr	0.2	0.4	0.2	0.3	0.2
1020	*p*-Cymene	-	2.2	0.2	0.1	0.4	0.6
1024	Limonene	0.8	7.4	2.6	0.7	7.1	5.3
1026	1,8 cineole	0.3	3.7	19.9	18.1	3.2	6.2
1032	(*Z*)-β-Ocimene	-	0.2	Tr	-	0.2	0.1
1044	(*E*)-β-Ocimene	-	1.1	0.1	-	0.9	0.5
1054	γ-Terpinene	Tr	0.3	0.6	0.3	0.5	0.4
1065	*cis*-Sabinene hydrate	-	0.2	0.1	0.1	0.1	0.3
1083	Fenchone	-	Tr	0.1	Tr	Tr	Tr
1085	Terpinolene	0.1	0.5	0.3	0.1	1.0	0.7
1088	*p*-Cymenene	-	Tr	-	-	Tr	Tr
1095	6-Camphenone	-	-	Tr	-	-	Tr
1098	*trans*-Sabinene hydrate	-	0.2	0.1	0.1	0.1	0.2
1100	Nopinone	-	-	Tr	-	-	-
1114	*endo*-Fenchol	-	Tr	Tr	Tr	Tr	Tr
1118	*cis-p*-Menth-2-en-1-ol	-	Tr	Tr	0.1	0.1	Tr
1122	α-Campholenal	-	-	Tr	-	-	Tr
1135	*trans*-Pinocarveol	-	-	-	Tr	-	-
1136	*trans-p*-Menth-2-en-1-ol	-	-	-	Tr	-	-
1141	Camphor	93.1	53.6	53.2	11.8	53.3	59.5
1148	Citronellal	-	Tr	Tr	-	Tr	Tr
1154	*trans*-β-Terpineol	-	Tr	Tr	Tr	Tr	Tr
1157	Sabina ketone	-	-	Tr	Tr	-	-
1160	Pinocarvone	-	Tr	-	Tr	-	-
1162	δ-Terpineol	-	Tr	0.4	0.3	Tr	0.1
1165	Borneol	0.4	0.3	0.4	0.4	0.7	0.7
1179	*p*-1,8-Menthadien-4-ol	-	Tr	Tr	-	Tr	Tr
1180	Terpinen-4-ol	0.1	1.2	2.4	1.5	1.4	1.1
1186	*p*-Cymen-8-ol	-	0.1	Tr	-	0.1	Tr
1187	Cryptone + Cymenol	-	-	-	Tr	-	-
1194	α-Terpineol	0.3	1.9	6.2	4.1	2.4	2.5
1197	Methyl chavicol	-	-	-	0.1	-	-
1199	*trans*-Piperitol	-	Tr	Tr	Tr	Tr	Tr
1204	Verbenone	-	-	-	Tr	-	-
1215	*trans*-Carveol	-	Tr	Tr	-	Tr	Tr
1223	Nerol	-	Tr	Tr	Tr	Tr	Tr
1227	Citronellol	-	0.1	0.1	-	0.1	0.1
1235	Neral	-	Tr	Tr	-	Tr	Tr
1237	Carvone	-	Tr	-	Tr	Tr	Tr
1248	Geraniol	-	-	Tr	-	-	-
1249	Piperitone	-	Tr	0.1	Tr	Tr	Tr
1264	Geranial	-	Tr	Tr	-	Tr	Tr
1282	Bornyl acetate	-	Tr	Tr	0.1	Tr	Tr
1285	Safrole	-	Tr	3.2	57.6	Tr	0.5
1332	δ-Elemene	-	Tr	-	-	Tr	Tr
1338	α-Santalal	-	-	Tr	-	-	-
1344	α-Cubebene	-	-	-	Tr	-	-
1345	Eugenol	-	0.1	1.0	0.4	Tr	0.2
1347	Neryl acetate	-	-	Tr	Tr	-	-
1348	Citronellyl acetate	-	-	Tr	-	-	-
1366	α-Ylangene	-	-	Tr	-	-	-
1374	α-Copaene	-	-	Tr	Tr	-	-
1376	Geranyl acetate	-	-	Tr	-	-	-
1388	*trans*-β-Elemene	-	0.2	Tr	-	0.4	0.2
1398	Methyl eugenol	-	-	Tr	0.1	-	Tr
1408	Dodecanal	-	-	Tr	-	-	-
1411	*cis*-α-Bergamotene	-	-	Tr	-	-	-
1416	α-Santalene	-	-	0.5	0.2	-	-
1417	β-Caryophyllene	Tr	1.1	-	-	2.3	1.4
1427	γ-Elemene	-	Tr	Tr	-	0.1	0.1
1431	*trans*-α-Bergamotene	-	Tr	0.1	Tr	Tr	Tr
1438	6,9-Guaiadiene	-	-	-	Tr	-	-
1444	*trans*-Isoeugenol	-	Tr	-	-	0.1	Tr
1445	*epi*-β-Santalene	-	-	Tr	Tr	-	-
1452	α-Humulene	-	2.7	0.1	Tr	0.3	0.9
1457	β-Santalene	-	Tr	0.1	0.1	Tr	Tr
1474	Selina-4,11-diene	-	Tr	Tr	-	Tr	Tr
1477	*trans*-β-Bergamotene	-	-	Tr	Tr	-	-
1480	Germacrene D	-	0.2	Tr	Tr	0.5	0.4
1487	β-Selinene	-	Tr	Tr	Tr	1.2	0.3
1492	α-Selinene	-	0.2	Tr	-	-	-
1505	β-Bisabolene	-	-	Tr	-	-	-
1509	Tridecanal	-	-	Tr	-	-	-
1513	γ-Cadinene	-	Tr	-	-	-	-
1516	δ-Cadinene	-	Tr	Tr	Tr	-	Tr
1517	Myristicin	-	-	Tr	0.3	-	-
1548	α-Elemol	-	Tr	Tr	-	Tr	0.1
1554	Germacrene B	-	Tr	Tr	-	0.1	0.1
1559	(*E*)-Nerolidol	-	Tr	Tr	-	Tr	Tr
1574	Germacrene D-4-ol	-	Tr	-	-	-	-
1577	Caryophyllene oxide	-	0.6	0.1	-	0.4	0.2
1582	Globulol	-	Tr	-	-	Tr	Tr
1592	Methoxy eugenol	-	-	-	Tr	-	-
1593	Guaiol	-	0.1	0.1	0.1	0.1	0.4
1598	*cis*-Bisabol-11-ol	-	0.1	Tr	-	-	Tr
1608	Humuleneepoxide II	-	0.7	0.2	Tr	Tr	0.2
1611	Tetradecanal	-	-	0.5	Tr	-	0.1
1621	Selina-6-en-4-ol	-	-	Tr	Tr	-	-
1623	1-*epi*-Cubenol	-	-	-	Tr	-	-
1640	*epi*-α-Cadinol	-	-	Tr	-	-	Tr
1650	Valerianol	-	-	-	0.1	-	-
1652	α-Cadinol	-	Tr	Tr	-	0.1	0.1
1658	Selin-11-en-4α-ol	-	0.7	0.1	Tr	0.1	0.3
Oxygenated monoterpenes		94.2	61.5	83.0	36.5	61.8	70.9
Sesquiterpene		Tr	4.5	0.9	0.5	4.7	3.3
Oxygenated sesquiterpenes		-	2.3	0.6	0.2	1.0	1.4
Phenylpropanoids		-	Tr	4.1	58.5	Tr	0.6
Other		-	Tr	0.7	0.1	Tr	0.2

**Table 3:** Enantiomer distributions of chiral terpenoid of *Cinnamomum camphora* L. essential oils.

Chiral Compound	Enantiomeric Distribution, Dextrorotatory (+) and Levorotatory (−)									
	Branch		Wood		Root		Leaf/Branch		Leaf/Branch/Wood	
	(+)	(−)	(+)	(−)	(+)	(−)	(+)	(−)	(+)	(−)
α-Thujene	70.3	29.7	30.9	69.1	7.3	92.7	75.0	25.0	70.9	29.1
α-Pinene	68.2	31.8	60.5	39.5	36.8	63.2	65.0	35.0	71.4	28.6
Camphene	59.8	40.2	70.0	30.0	0	100	48.4	51.6	62.5	37.5
β-Pinene	40.9	59.1	24.3	75.7	27.3	72.7	29.0	71.0	36.3	63.7
Sabinene	0	100	0	100	2.6	97.4	0	100	0	100
α-Phellandrene	99.1	0.9	93.5	6.5	83.6	16.4	97.7	2.3	98.2	1.8
Limonene	-	-	78.1	21.9	70.0	30.0	81.5	18.5	84.1	15.9
*cis*-Sabinene hydrate	-	-	16.5	83.5	8.2	91.8	64.8	35.2	66.7	33.3
*trans*-Sabinene hydrate	-	-	-	-	15.7	84.3	-	-	-	-
Camphor	99.5	0.5	99.6	0.4	99.0	1.0	99.6	0.4	99.5	0.5
Terpinen-4-ol	47.5	52.5	33.0	67.0	30.6	69.4	48.5	51.5	43.3	56.7
Borneol	72.2	27.8	0	100	0	100	70.1	29.9	73.6	26.4
α-Terpineol	70.5	29.5	23.4	76.6	10.1	89.9	86.4	13.6	71.2	28.8
β-Caryophyllene	0	100	-	-	-	-	0	100	0	100
Germacrene D	85.6	14.4	-	-	-	-	89.1	10.9	90.0	10.0

**Table 4:** Minimum inhibitory concentrations (MICs) of the *Cinnamomum camphora* L. essential oil against the tested bacterial strains.

Name of Bacteria	MICs (μg/mL)								
	*Cinnamomum camphora* L. Essential Oil						1,8-Cineole	(+)-Camphor	Safrole
	Leaf	Branch	Wood	Root	Leaf/Branch	Leaf/Branch/Wood			
*Bacillus cereus*	625	625	625	625	312.5	312.5	312.5	312.5	312.5
*Propionibacterium acnes*	312.5	312.5	312.5	312.5	312.5	312.5	625	625	312.5
*Pseudomonas aeruginosa*	625	625	625	312.5	625	625	312.5	312.5	312.5
*Serratia marcescens*	625	625	39.1	625	625	625	312.5	312.5	312.5
*Staphylococcus aureus*	1250	1250	1250	1250	1250	1250	312.5	312.5	312.5
*Staphylococcus epidermidis*	156.3	156.3	156.3	156.3	312.5	312.5	312.5	312.5	312.5
*Streptococcus pyogenes*	625	625	625	625	625	625	625	312.5	312.5

**Table 5:** Minimum inhibitory concentrations (MICs) of the *Cinnamomum camphora* L. essential oil against the tested fungal strains.

Fungal Strains	MICs (μg/mL)								
	*Cinnamomum camphora* L. Essential Oil						1,8-Cineole	(+)-Camphor	Safrole
	Leaf	Branch	Wood	Root	Leaf/Branch	Leaf/Branch/Wood			
*Aspergillus niger*	312.5	156.3	156.3	156.3	156.3	78.1	156.3	156.3	78.1
*Aspergillus fumigatus*	312.5	156.3	312.5	312.5	156.3	78.1	156.3	312.5	39.1
*Candida albicans*	312.5	312.5	312.5	312.5	312.5	312.5	156.3	156.3	156.3
*Microsporum canis*	312.5	312.5	312.5	312.5	312.5	312.5	312.5	312.5	312.5
*Microsporum gypseum*	312.5	312.5	312.5	312.5	625	312.5	156.3	312.5	312.5
*Trichophyton mentagrophytes*	156.3	156.3	156.3	312.5	312.5	156.3	156.3	312.5	312.5
*Trichophyton rubrum*	78.1	312.5	312.5	156.3	625	312.5	312.5	312.5	312.5

## Data Availability

All data are available in the manuscript.

## Supplementary Materials

The following are available online. Figure S1: Gas chromatogram of leaf essential oil of *Cinnamomum camphora* L. Figure S2: Gas chromatogram of wood essential oil of *Cinnamomum camphora* L. Figure S3: Gas chromatogram of root essential oil of *Cinnamomum camphora* L. Figure S4: Gas chromatogram of branch essential oil of *Cinnamomum camphora* L. Figure S5: Gas chromatogram of leaf/branch/wood essential oil of *Cinnamomum camphora* L. Figure S6: Gas chromatogram of leaf/branch essential oil of *Cinnamomum camphora* L.

## Sample Availability

Samples of the compounds are available from the authors.

## Abbreviations

**GC-MS**: gas chromatography-mass spectrometry, **MIC**: minimum inhibitory concentration, **EI**: electron impact, **DCM**: dichloromethane, **DMSO**: dimethyl sulfoxide, **CAMHB**: cation-adjusted Mueller Hinton broth, **CFU**: colony-forming unit.
