# Supplementary material for: The Chemical Profiling of Essential Oils from Different Tissues of Cinnamomum camphora L. and Their Antimicrobial Activities
Source: Molecules. 2021 Aug 24;26(17):5132. doi: 10.3390/molecules26175132 (PMC8434199; doi:10.3390/molecules26175132)
Supplement: Supplementary file 1 [file molecules-26-05132-s001.zip › molecules-1315915-supplementary.pdf]

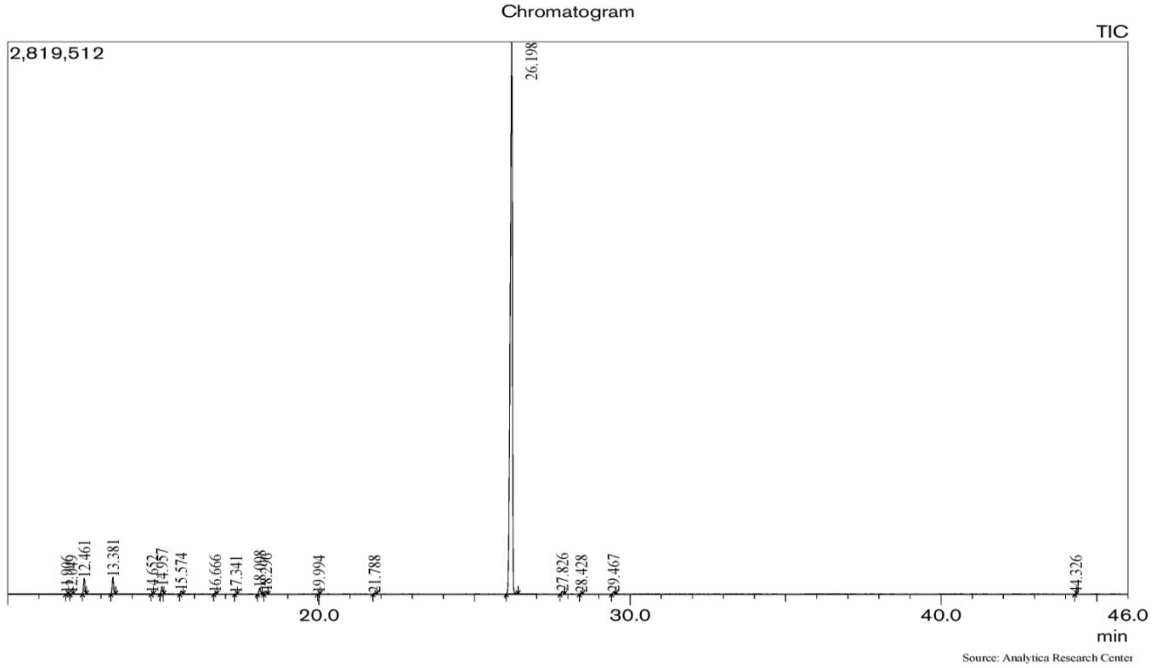

Figure S1: Gas chromatogram of leaf essential oil of *Cinnamomum camphora* L.

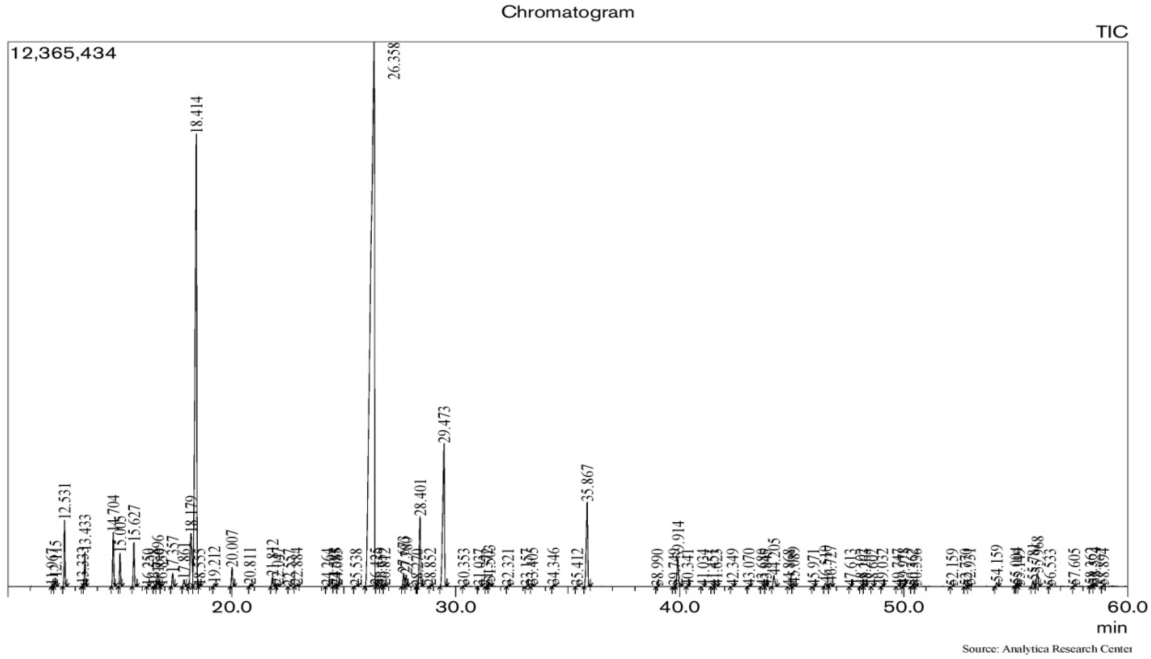

Figure S2: Gas chromatogram of wood essential oil of *Cinnamomum camphora* L.

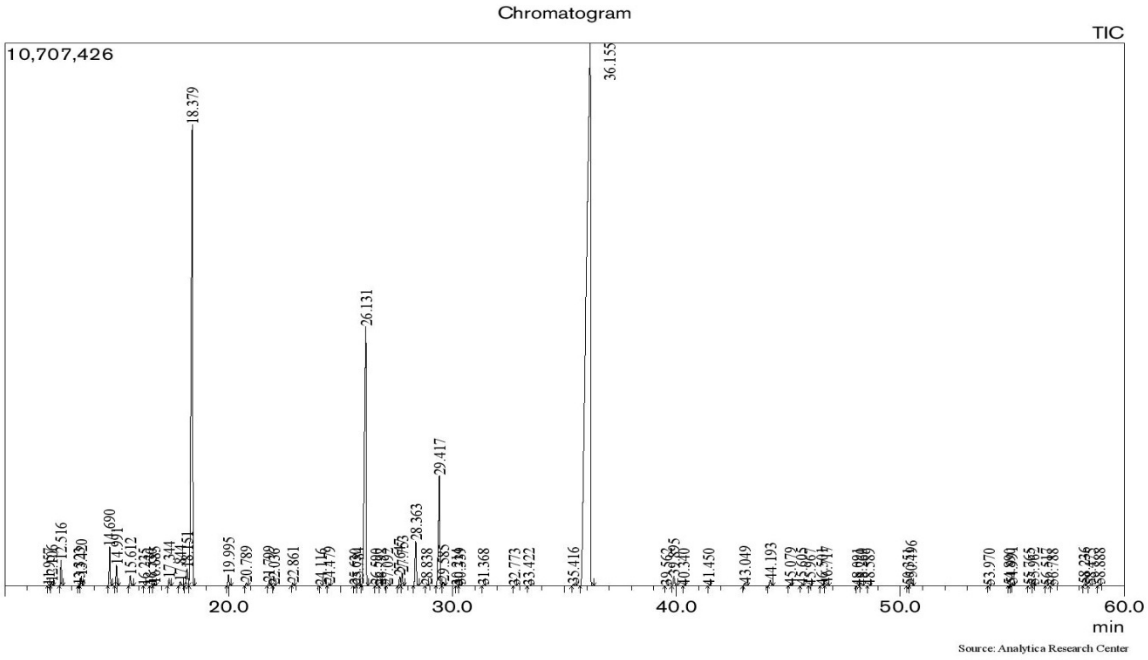

Figure S3: Gas chromatogram of root essential oil of *Cinnamomum camphora* L.

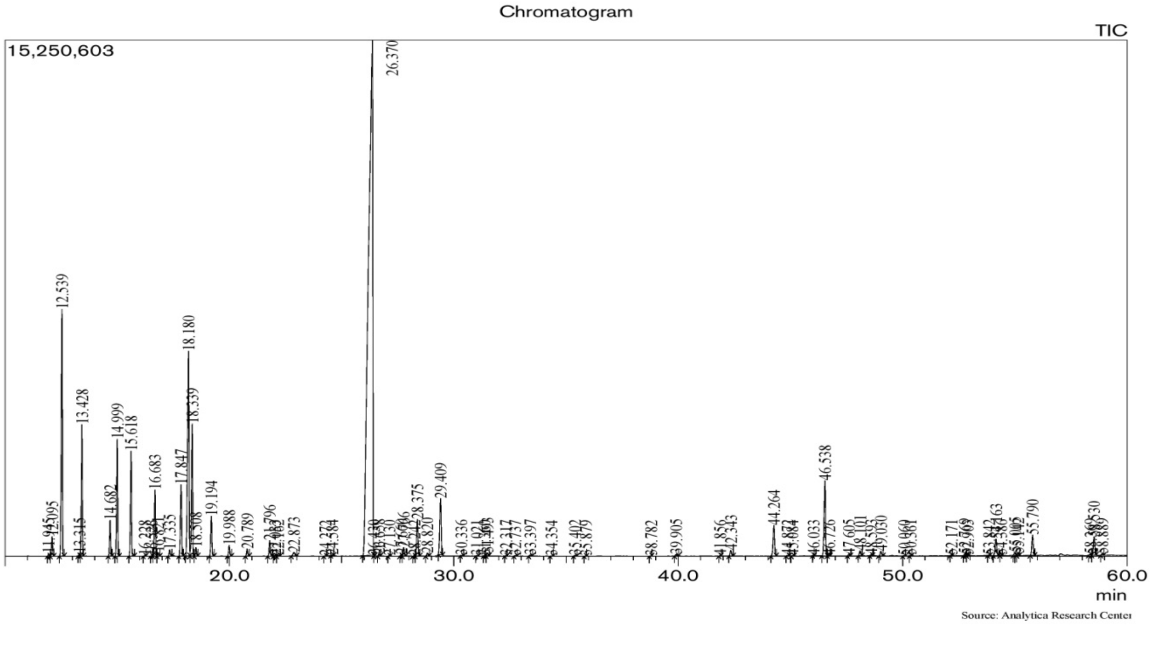

Figure S4: Gas chromatogram of branch essential oil of *Cinnamomum camphora* L.

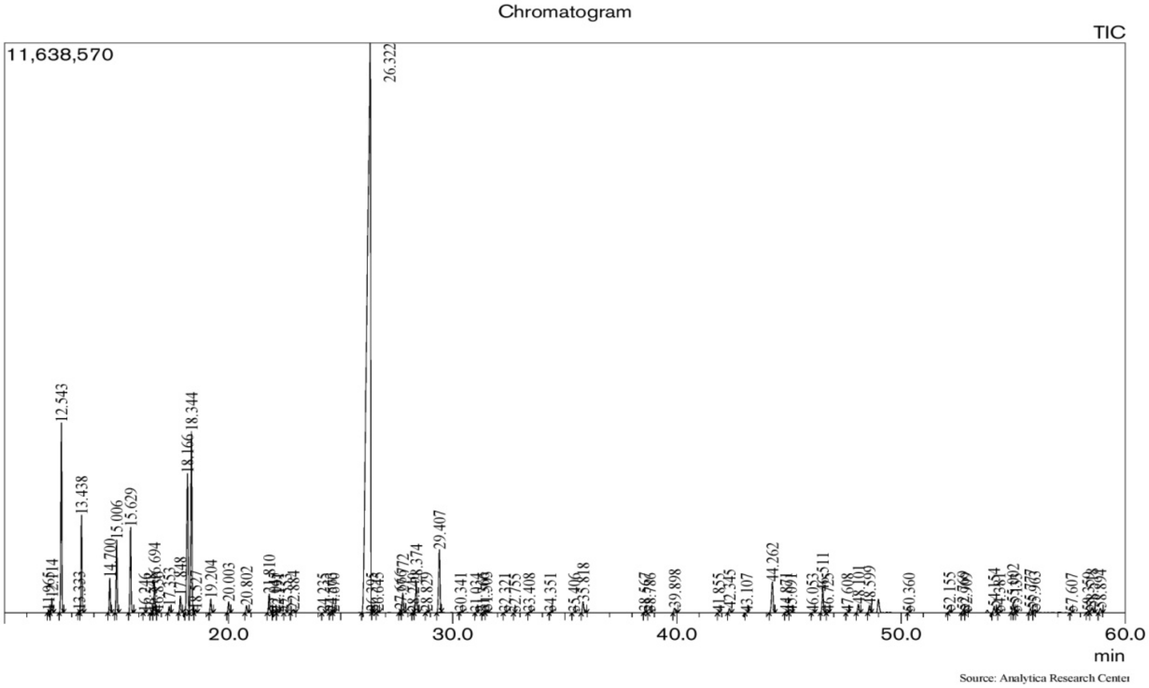

Figure S5: Gas chromatogram of leaf/branch/wood essential oil of *Cinnamomum camphora* L.

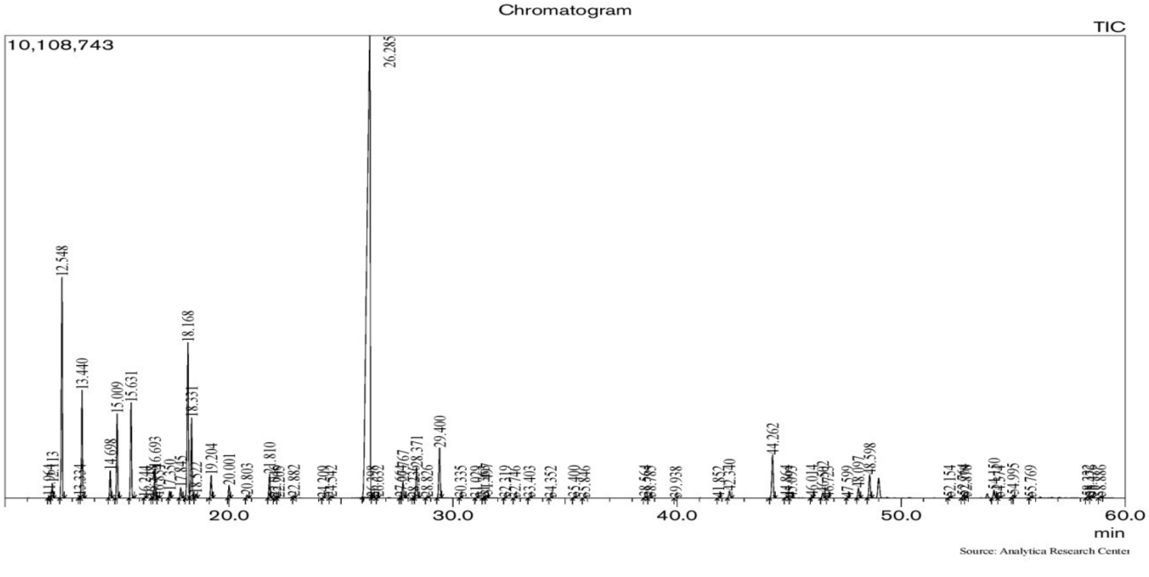

Figure S6: Gas chromatogram of leaf/branch essential oil of *Cinnamomum camphora* L.
